# Women’s Preference for Masculine Traits Is Disrupted by Images of Male-on-Female Aggression

**DOI:** 10.1371/journal.pone.0110497

**Published:** 2014-10-14

**Authors:** Yaoran Li, Drew H. Bailey, Benjamin Winegard, David A. Puts, Lisa L. M. Welling, David C. Geary

**Affiliations:** 1 Department of Educational, School, and Counseling Psychology, University of Missouri, Columbia, Missouri, United States of America; 2 Department of Psychology, Carnegie Mellon University, Pittsburgh, Pennsylvania, United States of America; 3 Department of Psychological Sciences, University of Missouri, Columbia, Missouri, United States of America; 4 Department of Anthropology, Pennsylvania State University, State College, Pennsylvania, United States of America; 5 Department of Psychology, Oakland University, Auburn Hills, Michigan, United States of America; 6 Interdisciplinary Neuroscience Program, University of Missouri, Columbia, Missouri, United States of America; University of Goettingen, Germany

## Abstract

Women’s preferences for men’s masculinized faces and voices were assessed after women (*n* = 331) were primed with images of male-on-male aggression, male-on-female aggression, pathogens, and neutral scenes. Male-on-male aggression and pathogen primes were associated with increased preference for masculine traits, but the same effect emerged in the neutral condition. We show the increased preference for masculine traits was due to repeated exposure to these traits, not the priming images themselves. Images of male-on-female aggression were an exception; these elicited feelings of disgust and anger appeared to disrupt the preference for masculinized traits. The results suggest women process men’s facial and vocal traits as signals of aggressive potential and lose any preference for these traits with cues indicating men might direct this aggression toward them.

## Introduction

Across species, females are predicted to prefer male traits that are reliable signals of genetic quality or the ability to provide direct resources [1]. Men’s facial and vocal traits may be examples of such signals in humans [2]. At this time, however, it is unclear if masculinized male traits evolved largely as signals to male competitors, as signals associated with female choice, or some combination [3]–[6]. Traits associated with dominance achieved through male-male competition may be signals of genetic quality but it does not necessarily follow that these same traits and underlying genotypes are always in females’ best reproductive interests [7]. Women often rate masculinized traits as more attractive than less masculine traits [8], but this is not always the case [3]. Recent studies have shown women’s preferences for masculine traits increase after viewing images of male-on-male combative competition (e.g., boxing) as compared to non-combative competition (e.g., golf) [9], consistent with a female preference for physically dominant males.

At the same time, the potential costs of mating with dominant and aggressive males have not been fully considered. Relative to their less masculine peers, men with masculine traits have more robust testosterone challenge responses and are behaviorally aggressive in competitive contexts, but may also be prone to aggression in the context of marriage or other long-term relationships [10]–[14]. These men may also be prone to sexual coercion outside the context of these relationships. Male sexual coercion of females is in fact found in many species [15], and humans are no different [16]); women engage in a variety of behaviors that reduce the risk of coercion especially by unfamiliar men (for recent review see [17]). The overall picture suggests cost-benefit tradeoffs whereby men with masculine traits have advantages over other men in physical male-male competition and the associated status may make them attractive mates, but at a potential cost of increased agonistic behaviors in the context of a marital relationship or heightened risk of sexual coercion outside of these relationships.

If women’s preferences are moderated by an implicit assessment of the cost-benefit tradeoffs associated with masculine traits, then viewing male-on-male aggression should enhance women’s preference for these traits, but viewing male-on-female aggression should reduce any such preference. Accordingly, we presented women with three types of violence primes: male intergroup aggression, male-on-male (one-on-one) aggression, and male-on-female aggression, and examined their influences on women’s preference for masculinized faces and voices. Control groups that received neutral primes or pathogen cues that will induce negative affect were also included [18]. We demonstrated that women have a preference for masculine traits when these are repeatedly presented but this preference is disrupted by exposure to male-on-female aggression. Their disrupted preference appears to be due in part to the emotions elicited when viewing these images.

## Materials and Methods

### Ethics Statement

The study was reviewed and approved by the Institutional Review Board of the University of Missouri. Written consent was obtained from all participants, and participants were debriefed after completion of the study.

### Participants

The participants were 331 female undergraduate students (M age = 18.5 years; SD = .8) who participated for partial course credit.

### Materials

#### Facial masculinity task

Twenty photographs of males with emotionally neutral facial expressions were used [19]–[21]. Each pair consisted of a masculinized and a feminized version of the same individual. Male and female prototypical (i.e., average) faces were first manufactured using established computer graphics techniques [22], [23]. These prototypes were then used to transform other images by calculating the vector differences between corresponding points on the male and female prototype images and applying a percentage of those differences to the corresponding points on a third image. The stimuli used in this study were manufactured by taking 50% of the linear differences between symmetrical versions of our male and female prototype faces and adding to or subtracting from the corresponding points on the facial images of the young adult Caucasian men (M age = 22.6 years, SD = 2.27) used in these studies [21].

In each trial, both the masculinized and feminized versions of the faces were presented side by side on a computer screen. The position (right, left) of the faces was counterbalanced across trials, and order of presentation across the 20 photo pairs was randomized across participants. Participants were asked to judge which male face was more attractive by pressing one of two keys (1 or 9) on the keyboard using the index finger of each hand. Once the participant responded, the face pair disappeared and the next pair was presented. For each trial, participants had 2 sec to respond.

#### Vocal masculinity task

Six Caucasian men (M age = 20.3 years, SD = 1.6) read the first sentence of a standard voice passage, the Rainbow Passage [24], using a Shure SM58 vocal cardioid microphone in an anechoic, soundproof booth (M = 5.3 sec, SD = 0.6). A curved wire kept each participant’s mouth approximately 9.5 cm from the microphone. Goldwave software was used to record voices in mono at a sampling rate of 44,100 Hz and 16-bit quantization; recordings were saved as uncompressed x.WAV files. Mean fundamental frequency (F0) was 110.6 Hz (SD = 15.7), and formant frequencies F1–F4 had means of 435.6 Hz (SD = 22.3), 1495.0 Hz (SD = 79.8), 2344.2 Hz (SD = 59.6), and 3322.9 Hz (SD = 112.4), respectively (see [25]). Both F0 and formant frequencies are highly sexually dimorphic (lower in men) and are the acoustic correlates of pitch and timbre, respectively [25]. Thus, in order to produce both masculinized (decreased F0 and formants) and feminized (increased F0 and formants) versions of the original voice files, we manipulated each recording in both acoustic parameters. F0 was manipulated by ±1.2 semitones, and formant frequencies were manipulated by ±4% using Praat v5.1.20 software. These manipulations correspond to intervals between masculinized and feminized stimuli of approximately two just-noticeable differences (JNDs) in both F0 and formant structure [26].

In each trial, participants heard both the masculinized and the feminized voice reading sequentially; the order of presentation of masculinized and feminized voices was counterbalanced across trials. Participants then rated vocal attractiveness by pressing keys (1 through 9) on the keyboard, with 5 indicating no preference, smaller numbers indicating a preference for the first voice, and larger numbers indicating a preference for the second voice. In each trial, the presentation of the first and second voice was separated by approximately 1.5 sec, and the total time for the presentation of both voices was exactly 14 sec. The next trial began when the participant responded.

#### Priming cues

Five categories of images were used as priming cues: male-on-male aggression (e.g., boxing), male intergroup aggression (e.g., armed soldiers), male-on-female aggression (e.g., a man slapping a woman), pathogen (e.g., a stained toilet), and neutral control (e.g., a man calmly reading). In a follow-up study of the order effect (see Results), participants only viewed the neutral control primes. Images were from the International Affective Picture System (IAPS) and were supplemented with images from an online search that were then validated using a separate sample [27]; (see Experimental Validation of Priming Cues in SI). Based on results from this validation sample, 40 pictures (26 from IAPS, 8 pictures in each condition) were selected for use in this study.

#### Emotion assessment

Participants reported their current emotional state on 5-point scale at the end of the study, with 1 indicating no emotional response and 5 indicating a strong emotional response. The assessed emotions were fear, disgust, anger, sexual arousal, romantic arousal, interest, tension, and happiness.

### Procedure

Participants were randomly assigned to one condition: male-on-male aggression (*n* = 67), male intergroup aggression (*n* = 64), male-on-female aggression (*n* = 68), pathogen (*n* = 64), or neutral control (*n* = 68). In the pre-exposure phase, participants completed the facial masculinity preference task (trials 1–20). Upon completing this task, participants were told that they would see a series of images on the screen and were instructed to look at each image carefully. After receiving these instructions, participants watched a set of 8 images presented in random order on the computer screen, and repeated 3 times. In each display, after a 100 ms orienting stimulus (+), a prime image was presented for 3 sec and then followed by a 100 ms blank screen. Immediately after the priming images were presented, participants completed the same facial masculinity preference task used in the pre-exposure phase (trials 21–40). Then, participants were again shown the priming images displayed in a random order and completed the vocal masculinity preference task. Finally, participants reported their emotions. The total testing time was about 30 min.

## Results

A random intercept model (trials nested within participants) tested whether masculinity preference varied across time (pre- to post-prime), priming condition, and time by priming condition. All analyses of facial masculinity preference and detection (see Supplementary Study of Repeated Presentation of Faces in File S1) data included stimulus as a random factor, and all random intercept models of facial masculinity preference and detection were logistic.

**Figure 1:**
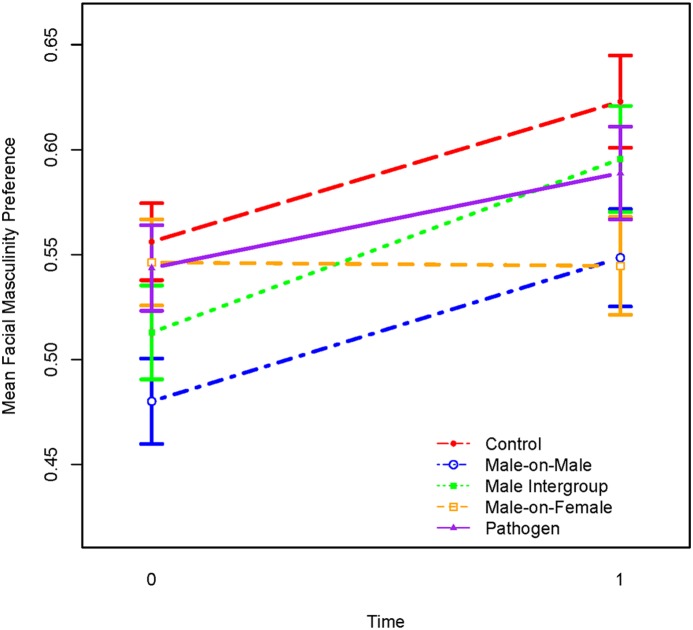
Pre- and post-prime (Time = 0 & 1, respectively) facial masculinity preference for each of the 5 conditions. Masculinity preference was evaluated by forced choice, with 1 indicating a preference for the masculinized face and 0 indicating a preference for the feminized face. Error bars are standard errors of the mean.

There were no group differences in masculinity preference at pretest (*F*_4,326_ = 2.06, *p* = .09). A main effect for time (*F*_1,326_ = 12.58, *p*<.001) indicated a pre- to post-prime increase in masculinity preference in the neutral control condition, and the interaction between time and priming condition (*F*_4,326_ = 3.02, *p* = .02) indicated that the change in masculinity preference differed by condition. Only the male-on-female aggression condition differed from the control condition (*p* = .011) (Table 1); specifically, the masculinity preference did not increase in this condition from pretest to posttest (Figure 1).

**Table 1:** Mean facial masculinity preference by time and condition.

	Estimate	SE	df	*t*-value	*p*-value
Intercept	0.56	0.02	326	25.81	<.001
Time	0.07	0.02	326	3.55	<.001
Male-on-Male	–0.08	0.03	326	–2.49	0.0134
Intergroup	–0.04	0.03	326	–1.40	0.1633
Male-on-Female	–0.01	0.03	326	–0.32	0.7461
Pathogen	–0.01	0.03	326	–0.40	0.6865
Time*Male-on-Male	0.00	0.03	326	0.06	0.9531
Time*Intergroup	0.02	0.03	326	0.59	0.5585
Time*Male-on-Female	–0.07	0.03	326	–2.57	0.0106
Time*Pathogen	–0.02	0.03	326	–0.80	0.4261

The neutral pictures condition is the reference group. Time is before versus after the primes. Therefore, time*condition effects are differences in the priming effect between the neutral and experimental conditions, and was only significant in the Male-on-Female aggression condition.

Because preferences for masculine faces increased with repeated exposure to these faces (i.e., an order effect) in 4 of the 5 conditions, including the neutral control condition, we suspected that presentation order, which was confounded with pre- to post-prime effects (i.e., time), created the appearance of a priming effect when such an effect might not exist. When masculinity preference was regressed on order and time (pre- to post-prime), there was a highly significant effect of order (*z* = 4.73, *p*<.001), but the effect of time was no longer significant (*z* = –.84, *p* = .40).

Therefore, we re-analyzed the data at the item level that allowed us to control for the effect of presentation order. In this model, the effect of order was significant (*z* = 4.74, *p*<.001) but not the effect of time (*z* = .05, *p* = .96), indicating that the neutral primes had no effect on masculinity preference above and beyond the repeated presentation of faces captured by the order effect. More importantly, the condition by time interaction remained significant for the male-on-female aggression condition (*z* = −2.68, *p* = .007), and was not significant for any other condition (all *z*<1, all *p*>.3). A follow-up study indicated the preference for masculine faces with repeated presentation may have been partially but not exclusively due to cross-trial improvement in the ability to detect masculine faces (see Supplementary Study of Repeated Presentation of Faces in File S1, Figure S1, Figure S2).

**Figure 2:**
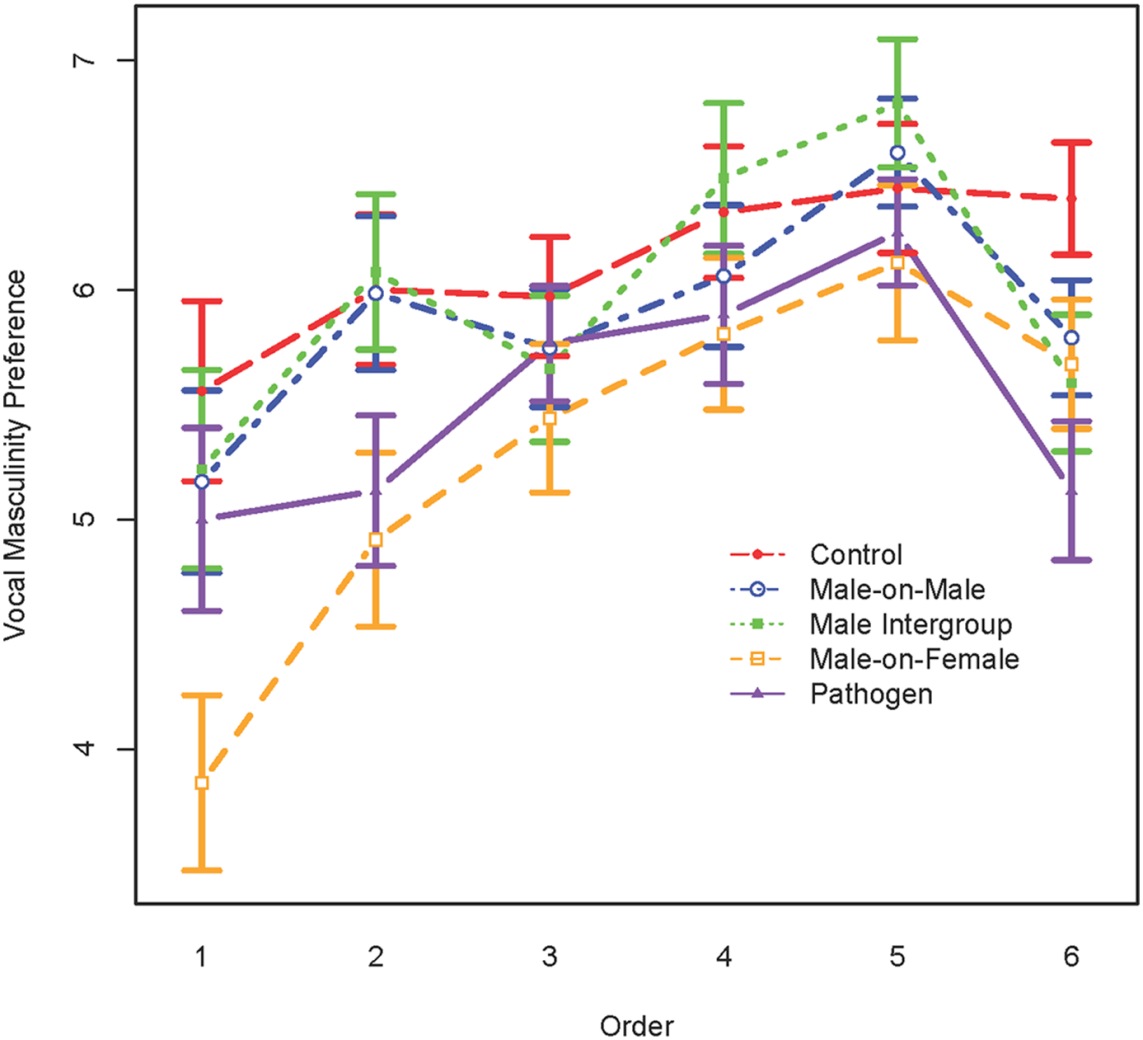
Vocal masculinity preference for each of the 5 conditions plotted across presentation order. Masculinity preference refers to rating on a 1–9 scale, with 1 indicating a strong preference for the feminized voice, 9 indicating a strong preference for the masculinized voice, and 5 indicating no preference for one voice over the other. Error bars are standard errors of the mean.

We then tested for the priming effect in the voice condition. The male-on-female aggression condition (*t*_326_ = −3.86, *p*<.001) was the only one that showed a main effect after controlling for order (*t*_1654_ = 6.09, *p*<.001). As with faces, following exposure to these primes, women had a significantly lower preference for masculine voices compared to women in the neutral control condition (Figure 2).

Next, we tested emotions as potentially related to the priming effects. We statistically controlled for these variables by adding them and their interactions with time to the model predicting masculinity preference from order, time, and time×condition.

For facial tasks, after controlling for disgust, anger, and their interactions with time, regression coefficient for the male-on-female aggression condition by time interaction was attenuated (–.23, compared to –.32 before the controls were entered) and was no longer statistically significant (z = −1.83, p = .07). However, the ratings for disgust and anger were positively skewed and thus we reran the analyses using their logarithm and observed the same results: a marginally significant interaction between the male-on-female violence condition and time (*p* = .06), and no significant interactions between time and the emotion variables.

Therefore, some combination of reported disgust and anger were associated with women’s decreased preference for masculine faces after viewing images of male-on-female aggression. However, reported emotions were not associated with the post-prime decline (relative to levels predicted by the order effect) in preference for masculine voices in this condition.

A supplementary study in which working memory load was systematically manipulated indicated that judgments of facial and vocal attractiveness were done without conscious, explicit decision making (see Supplementary Study of Working Memory and Face and Voice Processing in File S1). Moreover, we found the same order effects as in the main study and in the follow-up study (see Supplementary Study of Repeated Presentation of Faces in File S1). The results indicate that any demand characteristics of our procedures would not have influenced our findings.

## Discussion

The current study makes several distinct contributions to our understanding of women’s potential preference for masculinized faces and voices, although we note that the results only apply to faces and voices of unknown men and may have differed had we asked participants to rate the men as potential romantic or long-term partners. First, we found that presentation of images of male-on-female aggression disrupted their upward preference for masculine faces and voices across repeated exposures to these traits. The finding that this pattern was not evident for the two other categories of aggressive primes or for the negative affect pathogen primes indicates that images of male-on-female aggression were especially salient to our participants. The overall pattern is consistent with the view that any masculinity preference that women might have is moderated by the cost-benefit tradeoffs signaled by masculine traits and the processing of these tradeoffs occurs implicitly, without conscious decision making. These tradeoffs might reflect ambiguity regarding whether an associated tendency toward behavioral aggression during conflicts could be directed toward them (male-on-female aggression) or toward other males (male-on-male aggression) [10]–[12]. It is also possible that our primes elicited implicit concerns about sexual coercion [16], [17], independent of women’s mate choice tradeoffs. In any case, women reported feelings of anger and disgust after viewing male-on-female aggression and these emotional responses may be the implicit mechanism that disrupts their masculinity preference, at least for faces. The finding that these emotions were not related to their decreased preference for masculinized voices may have been due to the modality of the primes. If so, verbal male-on-female aggression may elicit these same emotions and disrupt a preference for masculinized voices.

Second, the finding that women’s preference for masculine traits increased with exposure, as suggested by our order effect in the main study and in the supplementary follow-up studies (see File S1), has implications for interpreting previous findings and for the design of future priming studies. With respect to interpretation, one potential explanation for the order effect is an improvement in participants’ ability to discriminate masculinized from feminized traits with practice. The results from our follow-up study (see Supplementary Study of Repeated Presentation of Faces in File S1) however, indicate that while this explanation is possible it is not likely. Another possibility is that the tasks themselves, with repeated exposure to masculine traits, primed an implicit perception of a skewed operational sex ratio, with abundant mating opportunities and a low level of competition (as no women’s faces were presented). This type of shift would, in theory result in an increase in selectivity of mate preferences, as found in some non-human species, such as the guppy (*Poecilia reticulata*; [28]) and St Peter’s fish (*Sarotherodon galilaeus*; [29]).

The results of a recent study are consistent with this interpretation. Watkins et al. [30] demonstrated that women’s masculinity preference is related to the sex ratio of viewed images. When women viewed photos with more men’s than women’s faces, their preference for masculinity increased and when they viewed more women’s than men’s faces their preference for masculinity decreased. However, these changes in masculinity preference were not statistically significant in Watkins et al. Also, women in the male-biased sex ratio condition were exposed to more men’s than women’s faces, thus it is uncertain which factor resulted in the increase: sex ratio or the overall number of men’s faces. Alternatively, Jones et al. [31] found that repeated exposure to attractive female faces resulted in an increased preference for attractive facial features in both men and women. Our results might then reflect a similar process, whereby women’s initial bias for masculine and presumably more attractive men’s faces is enhanced by repeated exposure, independent of sex ratio.

Finally, our findings suggests that pre-test to post-test difference in masculinity preference does not necessarily indicate a priming effect, since time (pre- to post-prime) is confounded with frequency of exposure, while a failure to find a pre-test to post-test difference in preference does not necessarily indicate a lack of a priming effect. Alternatively, it might be argued that our order effects were due to the length of the intervals between the presentation of faces and voices or other procedural details, but this would not explain the absence of the order effect in the male-on-female aggression condition. Furthermore, the same order effect emerged in the working-memory manipulation study (see Supplementary Study of Working Memory and Face and Voice Processing in File S1), indicating that these effects were not due to participants’ explicit awareness that masculinity preferences were being assessed. In any case, to draw firm conclusions about priming effects, it is critical to include a neutral-prime control group in the research design and to formally compare this group to the experimental group with order effects statistically controlled.

## Supporting Information

Figure S1 **Facial masculinity detection in follow-up study using only neutral primes.** Masculinity detection was evaluated by forced choice, with higher values indicating correct identification of the masculinized face (e.g., 0.9 = 90%). Order = stimulus presentation order; the vertical line indicates the priming manipulation.
(TIF)

Figure S2 **Facial masculinity preference plotted across the 40 face pairs and the order in which they were presented in the primary study (main text); the vertical line indicates the priming manipulation.** Masculinity preference was evaluated by forced choice, with 1 indicating a preference for the masculinized face and 0 indicating a preference for the feminized face.
(TIF)

File S1 **This file contains description of an experimental validation of the priming cues used in the main study.** Table S1–Descriptive statistics of pre-ratings of priming images; Supplementary study of repeated presentation of faces; Supplementary study of working memory and face and voice processing.
(DOCX)
