# Supplementary figures and images for: Women’s Preference for Masculine Traits Is Disrupted by Images of Male-on-Female Aggression
Source: PLoS One. 2014 Oct 14;9(10):e110497. doi: 10.1371/journal.pone.0110497 (PMC4197028; doi:10.1371/journal.pone.0110497)

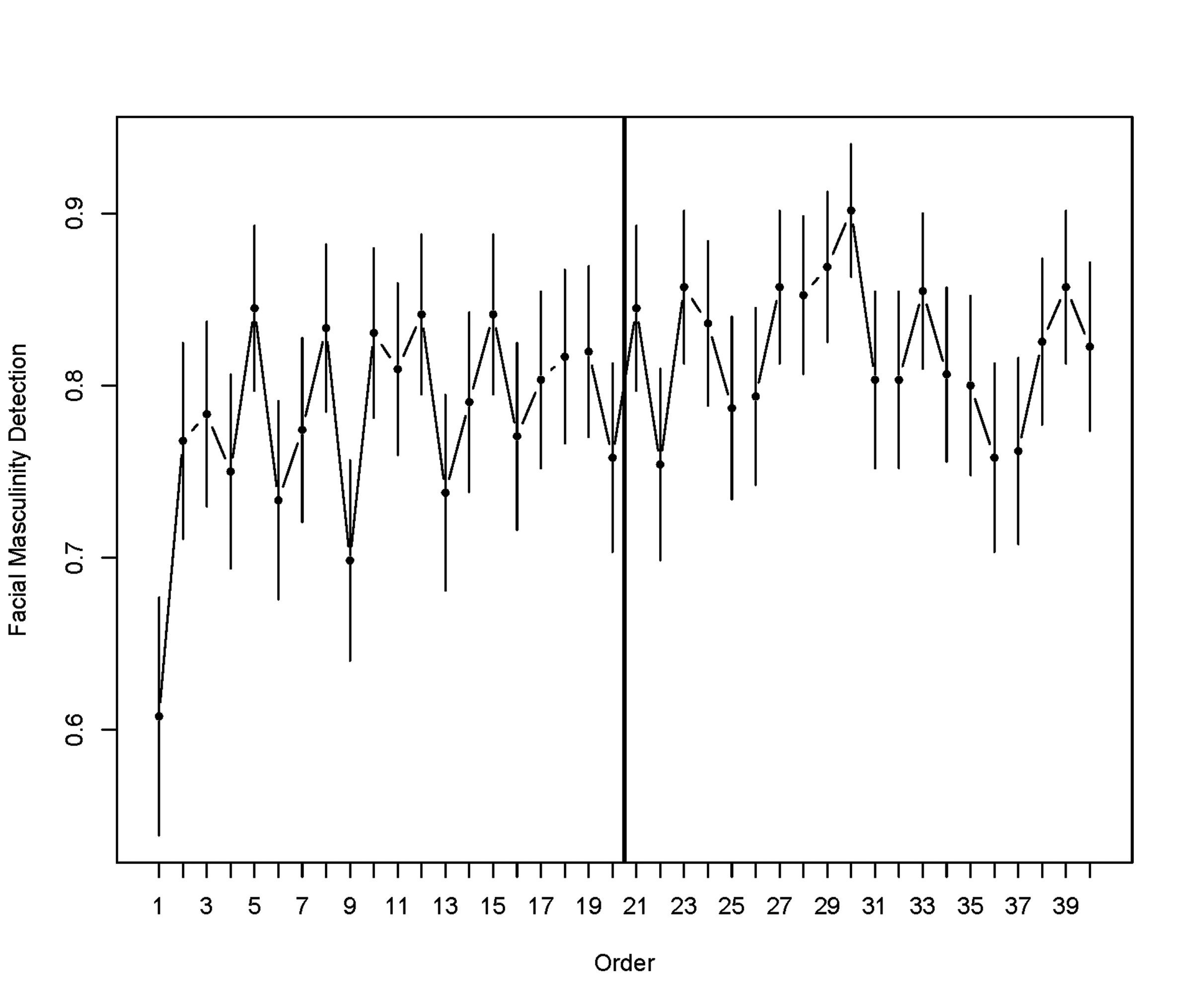

Supplement: Figure S1 — Facial masculinity detection in follow-up study using only neutral primes. Masculinity detection was evaluated by forced choice, with higher values indicating correct identification of the masculinized face (e.g., 0.9 = 90%). Order = stimulus presentation order; the vertical line indicates the priming manipulation. (TIF) [file pone.0110497.s001.tif]

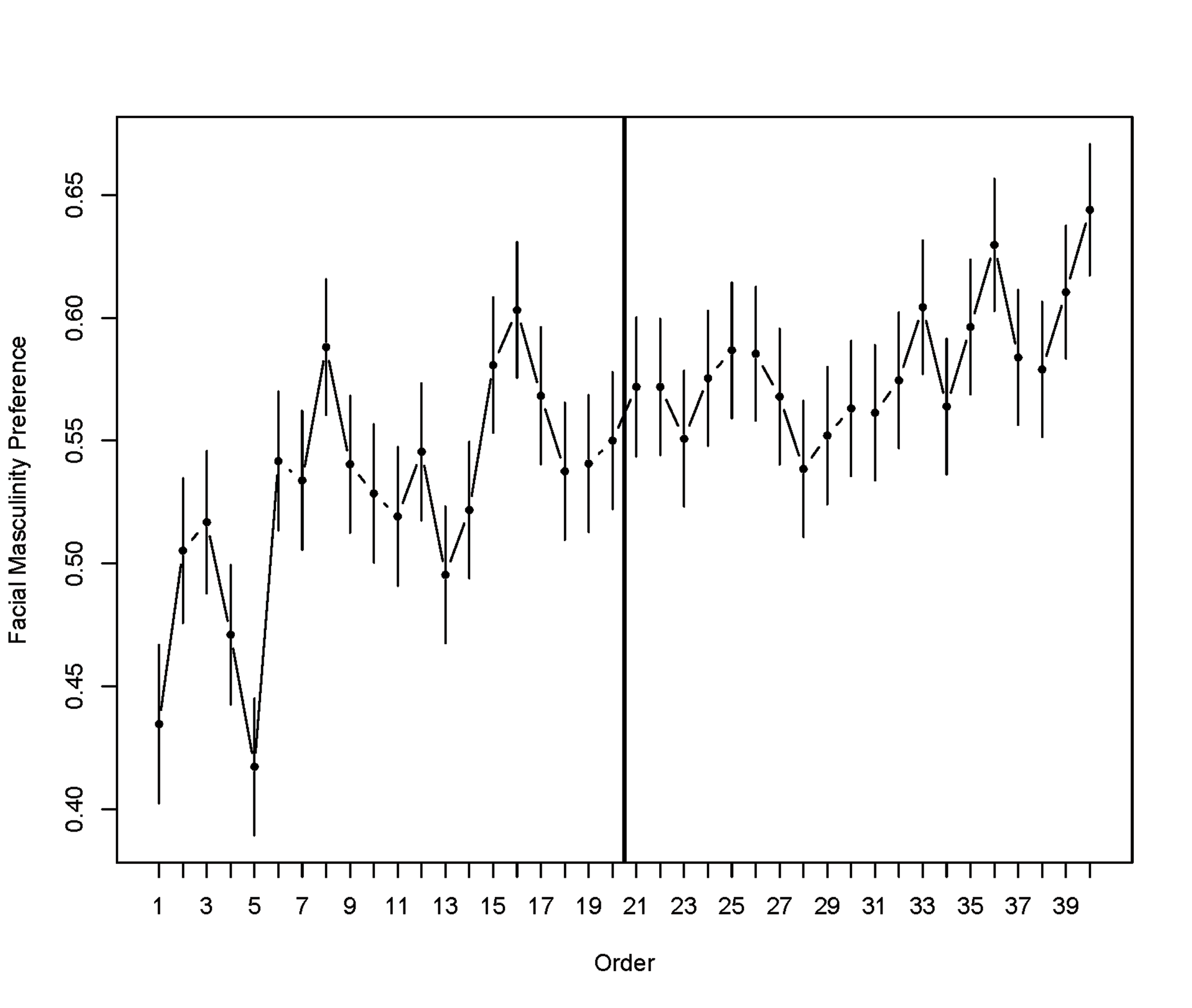

Supplement: Figure S2 — Facial masculinity preference plotted across the 40 face pairs and the order in which they were presented in the primary study (main text); the vertical line indicates the priming manipulation. Masculinity preference was evaluated by forced choice, with 1 indicating a preference for the masculinized face and 0 indicating a preference for the feminized face. (TIF) [file pone.0110497.s002.tif]
