# Supplementary material for: Women’s Preference for Masculine Traits Is Disrupted by Images of Male-on-Female Aggression
Source: PLoS One. 2014 Oct 14;9(10):e110497. doi: 10.1371/journal.pone.0110497 (PMC4197028; doi:10.1371/journal.pone.0110497)
Supplement: File S1 — This file contains description of an experimental validation of the priming cues used in the main study. Table S1–Descriptive statistics of pre-ratings of priming images; Supplementary study of repeated presentation of faces; Supplementary study of working memory and face and voice processing. (DOCX) [file pone.0110497.s003.docx]

**Supporting Information**

**Li et al. Women’s Preference for Masculine Traits is Disrupted by Images of Male-on-Female Aggression**

**APPENDIX S1: Experimental validation of priming cues**

To validate that the images elicited affective responses and arousal, 56 undergraduates who did not participate in the primary study (main text) rated the affect (positive vs. negative) and arousal (excited vs. calm) induced from each of 60 pictures on a 9 point scale; 34 were pictures of male-on-male aggression and for these images participants were also asked to determine if the conflict was group level or one-on-one. A higher score on the affect scale indicates greater negative affect and a higher score on the arousal scale indicates less arousal. Based on their ratings and norms for the IAPS [1], 40 pictures (26 from IAPS, 8 pictures in each condition) were selected for use in this study. As shown in Table S1, neutral control pictures were rated as producing less negative affect and less arousal than pictures in any other category (*p*s < .0001). Pathogen and male-on-female aggression pictures were rated as producing more negative affect than the other picture categories (*p*s < .01), and pathogen pictures were rated as producing less arousal than male-on-female aggression pictures (*p* = .005). None of the other pairwise comparisons differed (*p*s > .25).

**Table S1:** Descriptive statistics of pre-ratings of priming images. [Images were rated on a 1 (positive affect or excited) to 9 (negative affect or calm) scale.]

|  | **male-on-male violence** | | **male-male intergroup**  **violence** | | **male-on-female**  **violence** | | **pathogen** | | **neutral control** | |
| --- | --- | --- | --- | --- | --- | --- | --- | --- | --- | --- |
|  | M | SD | M | SD | M | SD | M | SD | M | SD |
| Affect | 6.22 | 1.14 | 6.59 | .62 | 8.01 | .87 | 8.32 | .42 | 4.40 | .62 |
| Arousal | 3.86 | .29 | 3.84 | .55 | 3.23 | .88 | 4.38 | .78 | 6.98 | .31 |

**APPENDIX S2: Supplementary Study of Repeated Presentation of Faces**

Results of the primary study suggested a general increase in preference for masculine traits with repeated presentation of these traits. Here, we assessed whether this effect is due to improvement in the ability to discriminate masculine traits with repeated exposure. We only used faces in this study, because their simultaneous presentation and thus opportunity to directly compare features across the pairs provided a more straightforward test of the discrimination hypothesis.

The procedure and analysis was similar to primary study, except that we only had one group (the control group), and the dependent variable was masculinity discrimination (which face is more masculine) rather than attractiveness. As in the primary study, there was a significant order effect (*z* = 3.33, *p* < .001). However, after omitting the first 9 trials, this effect was no longer significant (*z* = 1.28, *p* = .20), indicating that masculinity discrimination increased within the first few trials, but did not continue to increase throughout the task, as it did in the primary study; after trial 9, women correctly identified the masculinized face 81% of the time.

To further test how much discrimination can account for the relation between preference and order, we first regressed mean masculinity preference, aggregated across participants, from the control condition (primary study) on order. The order effect remained statistically significant, *t*(38) = 3.50, *p* = .001, *r*^2^ = .24. When masculinity detection, aggregated across participants, from the supplemental study, was added as a covariate, it did not have a statistically significant effect on masculinity preference *t*(37) = .93, *p* = .36, and the effect of order remained statistically significant *t*(37) = 2.76, *p* = .009.

We plotted presentation order by masculinity discrimination (Figure S1), which shows a clearly different pattern than the order by masculinity preference of the primary study (Figure S2). There is no order effect after the prime (beginning at order = 21) on masculinity *discrimination*, while there was such an effect on masculinity *preference* in the primary study.

**APPENDIX S3: Supplementary Study of Working Memory and Face and Voice Processing**

Human decision making, including those related to potential mates, may be based on automatic processes and heuristics or through deliberate and conscious evaluation of options explicitly represented in working memory [2,3]. Most of the behavioral choices of non-human animals reflect the former processes [4], but surprisingly the issue has not been experimentally addressed in studies of human mate preference tradeoffs. The issue is important to our current findings because it addresses whether the women were making judgments about the attractiveness of masculine traits based on explicit decision making, or inferences about what we may have been testing, or whether their preferences reflect the operation of more automatic, implicitly operating processes. Accordingly, we experimentally manipulated working memory load and induced ‘ego-depletion’ (i.e., prior engagement in cognitively demanding tasks [5,6]) to reduce capacity for explicit decision making while women rated the attractiveness of men’s masculinized and feminized faces and voices, as in the primary study. Participants also completed a Faith-in-Intuition scale that assessed the extent to which they reported making their decisions based on ‘hunches’, that is, without explicit decision making [7].

The experiment included a large sample of 345 women, yielding an 80% power of detecting a .30 standard deviation effect for the ego depletion manipulation, as contrasted with a 1.07 standard deviation increase in masculinity preference across the repeated presentation of face pairs (i.e., we found the same order effect as reported in the main text). The ego depletion manipulation was orthogonal to the working memory load manipulation embedded in the tasks, and thus the overall power of the combined experimental manipulations and the inclusion of working memory span scores in the analyses would result in a power higher than .80 for detecting small to moderate effect sizes.

We show that women’s processing of men’s faces and voices does not differ across levels of working memory load, nor did they relate to scores on the Faith-in-Intuition scale. The former result indicates that women are processing the faces and voices used in our study without making explicit decisions, that is, their preferences are based on implicit processing of facial and vocal traits. We suspect that the finding of no relation between faith-in-intuition scores and preference for masculine traits reflects people’s difficulties in explicitly describing (as in our questionnaire) how they make decisions that are based on implicit, prepotent and presumably evolved processes.

**Working memory manipulation.** The cognitive resources available during the masculinity preference tasks were manipulated in two ways. First, a standard working memory load manipulation was embedded into the masculinity preference tasks such that some of the preferences were made under low working memory load and others under high load. The latter makes conscious, explicit decision making difficult. Second, participants were assigned to cognitive depletion or control conditions (below). Depletion involved completing two difficult and standard working memory tasks, Stroop [8] and operation span (OSPAN, [9]), prior to the preference tasks. The Stroop and OSPAN tasks also provided measures of individual differences in working memory capacity and in combination took about 25 min to complete.

During the facial and vocal masculinity tasks, participants first saw a randomly selected string of 1, 2, 4, 6, or 7 letters presented for 7 sec on a screen. Participants were instructed to remember the string of letters for later recall. Letters were selected from a list of 9 consonants: F, H, J, K, L, N, P, Q, and R. After this screen, participants completed two trials of the facial masculinity task followed by one trial of the vocal masculinity task. Then, participants were prompted to type the letter string they were asked to remember into the computer. Participants repeated this 5 times, until they had completed all 10 facial masculinity preference items and all 5 vocal masculinity preference items. Each participant completed a set of two facial masculinity trials and one vocal masculinity trial under each of the five levels of cognitive load (i.e., while remembering 1, 2, 4, 6, or 7 letters). To ensure that participants were actually storing the letters in memory, we only analyzed masculinity preference trials for participants who correctly recalled the letter string.

**Working Memory Tasks.** In the Stroop task [8], a standard measure of the inhibitory control component of working memory, participants were presented with a string of letters, some of which were color words, presented in different colors on the screen. They were instructed to only respond to the color and to do so by pressing a corresponding key on the keyboard. There were 112 congruent trials (physical color and color-word match), 48 incongruent trials (physical color and color-word do not match), and 48 neutral trials (colored letter string in a row of Xs instead of a color word). The task took about 5 minutes to complete, and was scored by subtracting reaction times on correct incongruent trials by correct neutral trials.

Participants also completed a computerized version of the OSPAN task. Participants saw an arithmetic problem on the screen (e.g., 1*2 + 1 = ?), and were asked to solve the problem as quickly as possible. Participants then saw one of the 9 letters used in the facial and vocal masculinity preference tasks (F, H, J, K, L, N, P, Q, and R), which they were instructed to keep in memory. On the next screen, a number was shown (e.g., 3), and participants answered whether or not that number was the correct answer to the arithmetic problem that was presented two screens previously. Then, the participants saw another arithmetic problem, followed by another letter, and another potential answer. After this process cycled three times for 3 to 7 iterations (i.e., after participants had solved between 3 and 7 arithmetic problems), participants were prompted to select the letters in the order in which they had been presented from a list of 12 letters on the screen. The order of the number of letters to be remembered was randomized for each participant, and each participant was presented with a total of 75 letters and 75 arithmetic problems. Ospan scores were the total number of letters recalled correctly by each participant. The task took about 20 min to complete.

**Faith-in-Intuition scale.** Participants completed the Faith-in Intuition scale [7], as a measure of their reliance on intuition in their decision-making. The 5 item scale includes questions such as “I believe in trusting my hunches” and “I trust my initial feelings about people” (α = .72).

**Procedure**

Participants were randomly assigned to the depleted (*n* = 178) or control (*n* = 167) conditions; in the former participants completed the Stroop and OSPAN tasks before the masculinity preference tasks and in the latter after the preference tasks.

**Results**

The analyses were done with the nlme package [10] in R [11]. Mixed effects regression, with ratings nested in participants, was used to test whether masculinity preference was affected by condition (depleted vs. control), presentation order, or cognitive load (the number of letters stored in memory while completing the preference masculinity task). Separate models were run for facial masculinity preference and vocal masculinity preference. Initially, all 2-way interactions and the 3-way interaction were included in the model. None of the interactions were significant, and thus only the main effects are reported.

Validity checks for working memory manipulations

Participants correctly recalled 3.17 (SD = 1.35) letters from the letter strings they were asked to remember during the masculinity preference task, and answered 94% of the arithmetic problems correctly during the OSPAN task. Number of items remembered was significantly related to OSPAN score (*r*_343_ = .21, *p* = .0001). Condition did not influence Stroop or OSPAN score (both *t*_343_ < 1.2, *p* > .20). However, participants in the depleted condition recalled significantly fewer letter strings during the masculinity preference task (3.49 vs. 2.87; *t*_343_ = 4.36, *p* < .0001), consistent with the hypothesis that the depletion tasks did in fact reduce their subsequent working memory resources.

Facial and Vocal Masculinity.

In separate models predicting facial and vocal masculinity preference, the effect of presentation order was highly significant (faces: *t*_1877_ = 6.04, *p* < .0001; voices: *t*_783_ = 8.59, *p* < .0001), with higher masculinity preferences occurring at later presentation orders. Neither cognitive load during the preference tasks (faces: *t*_1877_ = -.99, *p* = .32; voices: *t*_783_ = .51, *p* = .61) nor depletion (faces: *t*_307_ = .46, *p* = .64; voices: *t*_307_ = .47, *p* = .64) was significantly related to masculinity preference.

Because the individual differences variables (i.e., Stroop, OSPAN, Faith-in-intuition scores) were measured at different time-points during the study for the depleted and control conditions, we analyzed the effects of these variables separately by condition. For both facial and vocal masculinity preference, and for each condition, we compared a model with cognitive load, presentation order, OSPAN score, Stroop score, and Faith-in-intuition as predictors to a model with just cognitive load and presentation order. In the control condition, the model with the individual differences variables (e.g., OSPAN scores) did not significantly improve model fit (faces: *χ*^2^_3_ = 2.86, *p* = .41; voices: *χ*^2^_3_ = 1.12, *p* = .77). The same pattern held for the depleted condition (faces: *χ*^2^_3_ = 2.57, *p* = .46; voices: *χ*^2^_3_ = 5.45, *p* = .14). In all models, no main effect of OSPAN score, Stroop score, Faith-in-intuition score or their interactions with cognitive load were significant (faces: |*ts*_307_| < 1.18, *ps>* .23; voices: |*ts*_307_| < 1.26, *ps>* .20).

**SUPPLEMENTARY REFERENCES**

1. Lang PJ, Bradley MM, Cuthbert BN (2008) International affective picture system (IAPS): Affective ratings of pictures and instruction manual*.* Technical Report A-8. University of Florida, Gainesville, FL.

2. Gigerenzer G 2001 The adaptive toolbox. In G. Gigerenzer, R. Selten editors, Bounded rationality: The adaptive toolbox. Cambridge, MA: MIT Press. 37-50.

3. Tversky A, Kahneman D (1974) Judgment under uncertainty: Heuristics and biases. Science 185: 1124-1131.

4. Davis JN Todd PM 1999 Parental investment by simple decision rules. In G. Gigerenzer, P. M. Todd, & the ABC Research Group editors, Simple heuristics that make us. New York: Oxford University Press. 309-324.

5. Baumeister RF, Bratslavsky E, Muraven M, Tice DM (1998) Ego depletion: Is the active self a limited resource? J Per Soc Psych 74: 1252-1265.

6. Hagger MS, Wood C, Stiff C, Chatzisarantis NL (2010) Ego depletion and the strength model of self-control: a meta-analysis. Psych Bull 136: 495-525.

7. Epstein S, Pacini R, Denes-Raj V, Heier H, (1996). Individual differences in intuitive-experiential and analytical-rational thinking styles. J Per Soc Psych 71: 390-405.

8. Stroop JR (1935) Studies of interference in serial verbal reactions. J Ex Psych 18: 643–662.

9. Unsworth N, Heitz RP, Schrock JC, Engle RW (2005) An automated version of the operation span task. Beh Res Meth 37: 498-505.

10. Pinheiro J, Bates D, DebRoy S, Sarkar D, the R Development Core Team (2012) nlme: Linear and Nonlinear Mixed Effects Models (Version 3.1-103): R package.

11. Ihaka R, Gentleman R (1996) R: A language for data analysis and graphics. J Comp Graph Stat 5: 299-314.
